# British Pakistani Muslim Masculinity, (In)fertility, and the Clinical Encounter

**DOI:** 10.1080/01459740.2017.1364736

**Published:** 2017-09-14

**Authors:** Mwenza Blell

**Affiliations:** Department of Sociology, University of Cambridge, Cambridge, United Kingdom

**Keywords:** British Pakistanis, assisted reproductive technologies, ethnic minorities, infertility, masculinity

## Abstract

The experiences of men facing fertility disruptions are understudied. For British Pakistanis, the impact of infertility is heightened for women because of normative pressures to bear children. But what of men? I present data from in-depth interviews in North East England with infertile British Pakistani Muslims and relevant health professionals. British Pakistani men’s level of participation in clinical encounters and responses to diagnoses of male factor infertility must be understood in the context of kinship, the construction of Pakistani ethnicity in the UK, and the subordinated forms of masculinity which accompany this identity.

British Pakistanis have among the highest fertility rates in the UK, reflecting strongly pro-natalist values and stigma associated with both childlessness and having one child (Culley and Hudson 2009). Previous research has indicated that British Pakistani women may receive unsatisfactory health care, especially maternity care (Baxter 1997; Katbamna 2000), due to racism in the National Health Service (NHS) (Bowler 1993). Additionally, studies from varied parts of the world have found that women often disproportionately bear the stigma of childlessness even when men are the ones who are infertile (Inhorn 2003; Bennett 2012). The assumption that women’s bodies are responsible for infertility is remarkable considering that male factor infertility appears to account for the majority of infertility cases worldwide (Kumar and Singh 2015). It has been argued that in some cultural contexts, men are protected from the threat to masculinity posed by male factor infertility by everyone, from professionals to partners (Barnes 2014). Barnes (2014:8) has suggested that “[m]ale infertility provides a useful case study for exploring masculine themes because infertility prevents men from accomplishing the most hegemonic form of masculinity.” In this article, I explore the relationship between gender and engagement with infertility treatment, focusing on British Pakistani men facing disrupted fertility. My analysis includes the experiences of these men and perceptions of them by others, including their wives and various health professionals. In what follows, firstly, I describe the methods used in data collection and analyses, then provide background information about the context of the research, both in terms of the population of study and the health care system. These are followed by sections about the major themes emerging in data analysis (including communication problems, anger, conjugality, disengagement, and blame), and an unfolding argument about the role of emotion, (hegemonic and subordinated) masculinities (Connell and Messerschmidt 2005), and other relations of power that shape both British Pakistani men’s actions and others’ perceptions of these men.

## Methods

This article is based on a three-year project investigating infertility in the lives of British Pakistanis. The study was conducted using a multi-site ethnographic approach over two phases. The first phase was conducted between 2007 and 2009 in a cluster of towns in North East England and consisted of participant observation and 86 life history interviews with British Pakistani women and men of different ages and reproductive statuses, in order to understand how infertility might influence the reproductive stories of a wider range of people than those diagnosed with infertility. Recruitment and participant observation for phase I was carried out in community centers, Sure Start children’s centers,^1^ taxi ranks, Asian cloth shops, and via snowball sampling.^2^ It was difficult to purposively recruit childless couples in non-clinical settings because of the stigma and secrecy that surrounds infertility. However, numerous people who had encountered some kind of fertility disruptions were recruited by chance in phase I.

The second phase occurred in 2010 and involved participant observation and in-depth interviews with individuals or couples who were undergoing or who had undergone fertility treatment, as well as interviews with key informants. Participant observation and recruitment took place in an *in vitro* fertilization (IVF) clinic within an NHS hospital in North East England. The aim was not to sit in on consultations but to observe the life of the clinic and to meet British Pakistani patients. Six interviews were conducted with clinic staff, including two fertility consultants, two embryologists, a nurse, and a counselor. Fifteen British Pakistani infertility patients (six couples and three individual women) were recruited through the hospital. Patients were given written information in English about the study and had an opportunity to discuss the study with the researcher (MB) or a research assistant, and those who consented were interviewed in their homes. Other key informants in phase II included two local professionals with South Asian Muslim backgrounds: a GP and a social worker from an agency specializing in ethnic minority adoption and fostering. In both phases, research assistants of Pakistani origin interpreted when interviewees were not proficient in English. All interviews were audio-recorded with consent, transcribed, and then entered along with field notes into NVivo software. The data were then coded thematically, based on principles of grounded theory, in which theoretical insights emerge from the data rather than vice versa (Strauss and Corbin 1998). After coding, excerpts with themes relevant for an analysis focusing on men and masculinities were re-read and analyzed to develop insight on men’s experiences and others’ perceptions of men. In the text, all names of individuals and institutions are pseudonyms.

## Pakistanis in North East England

Migrants from what is now Pakistan arrived in the seaports of North East England in significant numbers from 1941; most then moved south to Bradford and Leeds (Dahya 1974). There have been several shifts in the (self) perception of Pakistanis in the UK, responding to key events such as Partition, the Rushdie Affair, the Bradford Riots, and the London bombings on July 7, 2005 (often referred to as 7/7). These events, and their impacts on local communities, have generally promoted an emphasis on religious identity among British Pakistanis (Lewis 1994; Jacobson 1998). More recently, the group has felt itself threatened by racism and Islamophobia from wider British society (Bolognani 2007; Mythen, Walklate, and Khan 2009).

From historical and interview accounts, experiences of violence have been integral to the experiences of Pakistanis in the North East. Panayi (1991) discusses violence and what he terms a “race riot” in 1961 in Middlesbrough, in which Pakistani restaurants, homes, and clothing shops were attacked. In the immediate aftermath of the attack, non-white residents of the city fled in large numbers to find safety with friends elsewhere in the North East. During my fieldwork, informants told many stories of their experiences of past and more recent racially motivated violence directed at Pakistani men. One Pakistan-born father of five, in his late forties, reported that things had improved over time and that there were fewer such attacks in recent years. This informant recalled violent attacks against British Pakistani men from the 1960s to the 1980s, commenting that, “before you couldn’t walk out, you would get all sorts of abuse; your windows put through, all sorts, but we used to put up with it.” He explained that over time the local community had increased in size and Pakistanis were targeted less often, apparently because of their greater strength in numbers.

All Pakistani participants in the study were first- or second-generation migrants from Mirpur district in Pakistan-administered Kashmir or the Pakistani province of Punjab, and all identified as Muslims. The profile of British Pakistanis in the region mirrors the profile of the region itself, which is profoundly economically deprived. The geographical area of this study includes neighborhoods among the most deprived^3^ in England (Department for Communities and Local Government 2015). Furthermore, within the UK, people of Pakistani origin are on average more economically deprived (Platt 2011) and have lower rates of educational attainment (Khattab 2009) than the white British population. It has also been observed that ethnic minorities, including Pakistanis, living in economically deprived areas are poorer than white British people in the same locales (Garner and Bhattacharyya 2011).

The study participants’ educational levels varied, including several with no formal education and a few who were educated to degree level (for a full breakdown, see Hampshire, Blell, and Simpson 2012a). Among this population, marriage and childbearing are universal expectations. Additionally, marriages are often transnational, arranged, and consanguineous (first cousin) with virilocal (joint family) or neolocal post-marital residence. As described elsewhere, interviewees expressed a desire for larger families than the UK average, with many participants reporting that four children (two boys and two girls) as the ideal (Hampshire et al. 2012a).

## Local health care context

In the field site, a range of treatments for infertility were available to heterosexual, cisgender couples free at the point of service via the NHS. This included free Assisted Reproductive Technologies (ART) including *in vitro* fertilization (IVF), often carried out via intracytoplasmic sperm injection (ICSI). Couples experiencing infertility needed to first consult with their general practitioner to gain a referral to the local hospital for assessment, after which they are offered treatment options based on their area of residence.

Assessment involved consulting with reproductive medicine specialists, known as consultants, which meant answering questions and undergoing various lab tests and physical examinations. The questions included a medical and social history to fulfill a statutory requirement to take account of the welfare of any child produced via ART, or any existing children, before offering treatment. The UK Human Fertilisation and Embryology (HFE) Act 1990 states that “No treatment services regulated by the HFEA (including intrauterine insemination, IUI) may be provided unless account has been taken of the welfare of any child who may be born as a result (including the need of that child for supportive parenting)” and suggests “serious discord in the family environment” would be a cause for concern. The act defines supportive parenting as “a commitment to the health, well-being and development of the child,” and directs medical staff to “take account of wider family and social networks within which the child will be raised” where concerns may exist (HFEA n.d.).

For cisgender heterosexual couples, both partners were expected to attend consultations, and the man was expected to produce at least one but possibly several semen samples. In some cases, the female partner was put on drugs (e.g., clomifene) to stimulate ovulation. In cases where the semen sample was disease free and contained live sperm, intrauterine insemination was carried out using this sperm. If couples were unsuccessful in achieving pregnancy through these first-line procedures, they were put on a waiting list for free IVF/ICSI. In the site where I was recruiting, couples with no biological offspring were entitled to three “fresh” cycles of IVF/ICSI. Each “fresh” cycle could potentially result in a further two “frozen” cycles if enough viable embryos were produced. This meant that couples could potentially receive nine free cycles if the consultants believed there was a chance of success. However, undertaking such a high number of attempts could take years, during which time the likelihood of success and the likelihood of further cycles being authorized by consultants would progressively drop.

## Pakistani couples, communication, and encounters with professionals

Most of my time during hospital-based fieldwork was not spent talking about Pakistanis. However, the ward staff knew that a major focus of my study was the experiences of Pakistani patients and that I was approaching these patients for interviews. This led hospital staff to comment on their experiences of Pakistani patients. Some staff were not actually sure who was Pakistani, Bangladeshi, or Indian, if they were new patients, but learned more about patients’ backgrounds over the course of treatment. Some of the consultants seemed more confident than other staff in describing Pakistani patients as a group, perhaps because they were Arab Muslims and thus more aware of cultural and religious differences among particular Muslim patient groups. Common and interconnected perceptions concerning Pakistani patients included observations of Pakistani men’s disengagement from treatment, language problems, and male patients’ apparent anger and resistance to diagnoses of male factor infertility.

British Pakistani men’s lack of engagement was reportedly characterized by a refusal to provide semen samples and failure to attend appointments with their wives after initial consultations. Research in Pakistan in the same time period reported that Pakistani men regularly assumed that their wives were to blame and therefore refused to submit to semen tests (Mumtaz, Shahid, and Levay 2013), indicating consistency between Pakistanis in the UK and in Pakistan. British Pakistani men’s failure to attend appointments was, however, interpreted by health professionals as the result of a lack of a strong conjugal bond. This, in turn, raised concerns about the welfare of children potentially produced through assisted reproduction. Hospital staff were aware of the custom of Britain-Pakistan transnational arranged marriages. This awareness may have (negatively) informed how they viewed these relationships, since the government has long treated such unions with suspicion, as examples of “sham” or forced marriages (Shaw 2006; Charsley and Benson 2012) that may be associated with health risks to offspring (Shaw 2009).

Hospital staff were also aware that British Pakistani couples often lacked English proficiency due to transnational arranged marriages. Couples often lamented what they felt was less respectful treatment within the NHS to people with limited English language skills. Hospital staff, however, raised concerns about relying on partners as interpreters, reflecting their suspicions about their relationships. One consultant questioned whether couples could be considered to be “genuinely married” if their “main bond is to have children... [rather than] personal bonding.” Language issues were entangled with questions over the nature of these relationships:

Consultant: They are quite shocked when you tell them your sperm is not good, then they start to either not attend…

Author: Not attending the appointment?

Consultant: Yes or delaying in getting the sperm analysis or they produce it once and they don’t want to repeat it – not all of them but this is the majority. And they try to leave the lady herself to come in. Some of them, yes, they may come… it’s very personalized when you tell them that [their] sperm is not good. But it seems that effect [is] not happening when you tell the woman they are not producing eggs… the reaction is different, as if you were insulting the man in some way.

… and the problem we’ve found, if they work as interpreter to the ladies… That’s why we insist to get them in turn, because they may not pass all the information and they try to reverse it as blaming one party, which is the female, as she is the person who is not getting pregnant. They don’t look at it as a problem for the couple…

These comments reflect a narrow view of what constitutes a legitimate marriage, one at odds with the practices of many of my British Pakistani research participants and their families. Jamieson (2012) notes that in European and North American cultures, the importance of romantic love and intimacy in relationships is taken for granted, reflecting assumptions of individualism which may not be shared more widely. de Kok’s (2013:35) work on infertility in Malawi has highlighted the importance of considering “how people themselves orient to relationships” rather than assuming what constitutes discord and a threat to marriage. On the basis of her work with infertile couples in Indonesia, Bennett (2012:7.2) similarly argues that “it is important not to be blinkered by a culturally and historically specific understanding of intimacy.”

As my interviews with hospital staff indicated, not all patients spoke English very well or at all. Nurses did not think that this was a big problem. In some instances, one partner interpreted for the other; occasionally a professional interpreter was provided by the NHS; in other cases, couples would bring along a lay interpreter. One nurse said that for Pakistanis there was “always some way to get the information to the couple,” in contrast to newer Eastern European migrants, for whom it was not always possible to find an interpreter. Although there is suspicion of Pakistani husbands’ interpreting, this suspicion is not typically handled by bringing in professional interpreters to prevent problems.

One nurse recounted the story of a Pakistani couple dealing with male factor infertility who had deliberated for a long time over whether to use donor sperm. The hospital staff were aware of a general rejection of donor sperm by South Asian Muslim couples, as previously documented (Culley and Hudson 2007). However, this nurse said that the couple, who did not speak much English, finally agreed to the use of donor sperm and came to the hospital for their insemination appointment. It became clear when preparing for the procedure that the couple’s reluctance to use donor sperm was because they understood the method of insemination to be sexual intercourse with the donor. Clearly, communication problems can cause considerable unnecessary anxiety.

Communication problems in clinical consultations about infertility were not limited to language fluency, and occurred even when one or both parties were native speakers of English. Some couples on the IVF waiting list did not know much about IVF, and used their interviews with me to gain further information. They typically wanted to know what would happen when they reached the top of the waiting list and how likely the treatment was to be successful. Lack of sufficient knowledge of the treatments, particularly given the widespread misconception that treatments necessarily involved donor gametes, partly explained the disengagement or the negative emotions of men in consultations. Since IVF information sessions in the hospital were not provided for non-English speakers, some could not access the sessions at all. For couples in which one partner had better knowledge of English, this partner was relied upon to interpret information in the session. Their translations may lack accuracy because of the difficulty of translating medical terminology and concepts, rather than the difficulty of communicating in a second language.

## Masculinity and anger

Consultants, nurses, a local GP (of Pakistani origin), and one of the embryologists interviewed all referred to anger and/or resistance to diagnoses of male factor infertility and a tendency to blame wives; they were, one consultant described, “not cooperative,” at times leading to disengagement from treatment. The GP explained:

It’s very difficult… it takes [a] long, long time for things to – they find it very difficult that a man could be deficient of something. They find it very difficult to accept, for them, that there is something wrong with them [and] that’s why a child can’t be born.

Connell and Messerschmidt (2005:832) have described hegemonic masculinity as “the currently most honored way of being a man” in a particular cultural and historical context, requiring “all other men to position themselves in relation to it.” Drawing on cross-cultural data, Dudgeon and Inhorn (2003) describe hegemonic masculinities as incorporating, among other things, wealth, virility and emotional detachment, and have noted that failure to achieve such ideals of behavior may be distressing. British Pakistani men living in economically-deprived North East England, with the sustained threat of Islamophobia and racism, and facing disrupted fertility, are intersubjectively positioned at a distance from the ideal of hegemonic masculinity in British society. These men are associated instead with particular subordinated masculinities inflected with postcoloniality, racism, and Islamophobia, and this influences their positioning and behavior when seeking infertility care. Failure or temporary delay in becoming a father can also be seen as a disruption in achieving what is expected of any successful adult Pakistani man.

Kalra (2009:119), in his discussion of British South Asian masculinities, points out that “the current focus on Muslim masculinities is irrevocably linked to the policy terrain marked by ‘home grown-terrorists’, 7/7 and the perceived threat of Islam.” Alexander (2004) has described perceptions of British South Asian men as angry, constructed within “folk devil” categories including gang member and terrorist that position them as outsiders posing a threat to broader society. Mac an Ghaill and Haywood (2005, 2015) show how young British Pakistani and Bangladeshi Muslim men enact multiple masculinities by relating in varying ways to tradition and religion. However, this work also highlights the effects of racialization, racial exclusion, and Islamophobia on Pakistani men, who are simultaneously positioned as terrorists and as potential victims of radicalization.

British imperialism constructed racial typologies of its subjects in which Muslim men from the region which is now Pakistan were commonly described as members of the “martial races,” endowed with stupidity (particularly poor abilities in terms of education), a barbaric tendency to violence, and an obsession with honor (Omissi 1991; Chowdhry 2013; Rand and Wagner 2012). These notions served to legitimize colonial control of such dangerous and unintelligent men and their inclusion among the disciplined ranks of the colonial army (Omissi 1991; Chowdhry 2013; Rand and Wagner 2012). Considering this, it is not surprising that Pakistani men’s reactions to clinical consultations about infertility are read as expressions of anger. Ahmed (2004:119) suggests that emotion need not be considered to “positively reside in a subject or figure” but can be thought of as merely seeming to be located within a person because of a historical association. Anger, in this case, “opens up past histories that stick to the present” (Ahmed 2004:126) and is crucial to understanding how history has shaped this particular form of subordinated masculinity.

On one occasion during fieldwork, I travelled by taxi to the home of a Pakistan-born couple I had met briefly in the hospital, planning to interview them. I had arranged the meeting in advance and when the taxi pulled up to the house, the male interviewee was standing outside. He was a large man, wearing a beige *shalwaar kameez* (a traditional South Asian outfit consisting of loose trousers and a long tunic) marking him out as Asian, and he was wearing what I read as a foreboding expression. The white British taxi driver read his expression in the same way, and spoke to me in a low voice, asking if I thought I was safe or whether I wanted him to drive on. I reassured the taxi driver, despite my own sense of apprehension, and got out of the taxi. Neither member of the couple spoke much English, and both were hospitable and very willing to talk. I found the husband’s willingness to sit with his wife and talk with us surprising, because his expression still seemed angry. Moreover, many Pakistanis consider it good manners for men to absent themselves when there is a female guest. I discovered during the conversation that the husband had an injury that caused constant pain, and realized that his facial expression reflected physical pain not anger. Wearing shalwaar kameez has long symbolized difference in relations between British Pakistanis and the wider society (see Dahya 1965 for an early account) and would emphasize Asianness to strangers such as the taxi driver. Were the taxi driver and I more willing to read the expression as anger because of how he was dressed? Might others be misreading pain for anger when they encounter men they know or assume to be Pakistani? Infertility is often described as emotionally distressing, and it is increasingly recognized that social and emotional pain are not just metaphorical, but are physically experienced as pain (Eisenberger and Lieberman 2004).

For Pakistan-born men, language and communication problems may also play a role in inducing frustration that is subjectively experienced as anger. One Pakistan-born couple I interviewed twice experienced no resolution to their fertility problems, and the wife was continually experiencing distressing gynecological symptoms. They felt trapped by their poor English proficiency and discriminated against by the NHS. The husband expressed his frustration as follows:

Can you tell me which kind of life you call this one? … What kind of life does she get? I feel like going to the hospital to smash his head in.… We can’t do anything else because we have no money, we have no skills, for these kinds of things you need skills and only me and my wife… I can’t write my name or my address or date of birth, if someone asked me can you write a small [letter]… I can’t write.

This quote demonstrates intense feelings of powerlessness and frustration on the part of the male partner, who lacks the “skills,” “money” and social capital required to navigate fertility services. Such feelings of powerlessness for Pakistani men are amplified by the reality that men face a dual struggle in terms of performing masculinity. To achieve Pakistani ideals of successful adult masculinity, men must become fathers and producers of the next generation of the extended kin group (Charsley 2005). Infertility directly threatens the cornerstone of this relational and family-centered form of masculinity. At the same time, many Pakistani men are excluded from attaining markers of hegemonic masculinity in UK society, due to their lower socio-economic status and continued racism. Charsley (2005) has also described observing anger and aggression as reactions to threatened masculinities among young Pakistan-born husbands in the UK. Expressing distress and pain as anger may be thought of as less emasculating than other emotional responses, but would seem problematic for British Pakistani men, playing into the negative views of them as a group.

When asked about the emotional side of their experiences of infertility, men said little. The following exchange with a British-born couple in their thirties is typical in its economy of expression:

Author: Do you think it’s been harder on one of you than the other?

Husband: I do, on me.

Author: You think it’s been harder on you?

Husband: Yeah, I can’t say why but I answered your question.

Wife: Why because you’re older or–?

Husband: [*with heightened emotion*] No, it’s not ‘cos I’m older, it’s my fault, isn’t it? …The only thing that I wished was, I wished they had found [sperm] but they have done everything and you can’t go against the system.

Wives spoke more about the emotional impact on their husbands than husbands did, while at the same time praising their partners’ masculine responses. Pakistan-born Javaid said nothing about his emotions in an interview with the couple, but when left alone to speak to me, his British-born wife Rukhsana explained:

I could never forget it because it put me down, when [the doctor] got the results of my husband’s sample, he just looked at me and he looked at the computer and he was like [*shakes head*]

Author: He just shook his head?

Rukhsana: I thought, ‘oh my God, what does that mean?’ and my husband he nearly sank to the floor… My husband is upset about it but he is strong and he has never ever let it get to him, he’s been an inspiration to me as well… even if I’m just sat there watching something and a baby comes along and I’m like, ‘ah, so sweet’ and he’s say ‘yeah, we’ll have a baby like that as well’… so he is really, really strong, he was really upset when we got the results but after that he has been fine.

As she shares details of Javaid’s distress, Rukhsana presents him in a manner that emphasizes masculine characteristics such as his strength and ability to control his emotions. Thus, my observations of the emotional expectations of Pakistani couples were that the male partners were expected not to overtly show or express the vulnerability or distress they experienced as a result of infertility. Silence, stoicism, or even frustration are understood as culturally acceptable responses for men. Such responses affirm acceptable masculine behavior for Pakistani men within the context of their marriages and communities.

## Conjugality, blame, and disengagement

This discussion of emotion leads to a consideration of the extent to which infertile couples were focused on their conjugal relationship or, in the consultant’s words, whether they were “genuinely married.” Walle (2004:129) notes that Pakistani men in Lahore view “romantic love as unmanly or feminised” and that such emotions would not be publicly expressed. This cultural norm may strengthen health professionals’ perception that conjugal bonds among Pakistani couples are weak. Wider family networks, especially with older relatives (aunts, uncles, grandparents), also influence conduct in marital relationships, including reproduction. Elders are often mindful of “the community” observing marriages and gossiping about young couples. The norm of virilocal arranged marriage can make wives more vulnerable to criticism from the husband’s family. Additionally, the permissibility of polygyny (but not polyandry) within Islam means that a woman’s security within marriage can be destabilized by infertility.

Elsewhere, I have illuminated the relationship between education, financial independence, and an emphasis on the conjugal relationship among British Pakistani couples (Hampshire, Blell, and Simpson 2012b). Couples who are more educated and financially independent, often but not always with a British-born male partner, are sometimes able to build stronger conjugal relationships. Couples in stronger relationships tend to be more resilient in the face of pressure from the wider kinship network to have children quickly. Couples with limited education and financial independence may have greater reliance on kin networks and be more subject to family pressures. This is evident in the case of Naseem and her husband, both born in the UK, who had difficulty conceiving due to male factors. Naseem’s account of married life was full of references to limited family resources and her mother-in-law’s anxieties about any expenditure on her or her husband’s part. Naseem’s mother-in-law discouraged and disrupted attempts by Naseem and her husband to spend time alone together, asking: “What are you talking about? … Boys don’t stand in the kitchen and talk to their wives.” Naseem’s mother-in-law tried to enforce the idea that marriage does not include the practices of intimacy, which might be considered central to relationships and family practices in the West (Jamieson 2012). In this alternative view of marriage, the most important relationship practices are procreating.

Whilst this couple was eventually able to conceive, Naseem’s husband resisted treatment for a long period, refusing to provide semen samples or attend consultations. During this period, Naseem was blamed by her in-laws for the failure to conceive, taken for multiple consultations with nonbiomedical healers, and forced to take unpleasant herbal medicines. Eventually, she resisted:

I put my foot down and told him… ‘I’m not taking them, it’s my body’, I said ‘there’s nowt [i.e. nothing] wrong with my body, it’s you’… ‘I’m not taking any more of this crap, I don’t care what they say… I said ‘I know the problem’s probably you, it’s from, taking steroid drugs and that and bodybuilding’ and whatever he was doing and he’s – he did stop and then I, like I said, I fell [pregnant].

Naseem’s case is one of many in which in-laws loomed large in the development of the conjugal relationship and the bearing of children. Some women, like Naseem, described their opportunities to develop closer relationships with their husbands as blocked by in-laws, particularly mothers-in-law and sisters-in-law. At the same time, women felt under pressure to become pregnant, paradoxically, to solidify the marriage. However, other couples, equally young, were explicitly encouraged by family elders to take time to get to know one another and not rush to have children.

Concerns about money, status, and reputation within wider kinship networks provide some explanation for the motivations of elders. The major motivation for migration to the UK from Pakistan is the desire to increase power and prestige for patrilineages, known as *biraderis* (Werbner 1990). Production of offspring of both sexes is important to continue the patrilineage, especially in the context of transnational arranged consanguineous marriages, as brides with British passports or residency are needed to bring over their cousins from Pakistan as grooms (Charsley 2005). These kinship relations involve traditionally competitive elements, documented by numerous ethnographers (Barth 1959; Ahmed 1980; Ballard 2003; Lyon 2004). Men compete with their fathers’ brothers’ sons for any property belonging to their grandfathers (Lyon 2004), and as Ballard (2001) has observed, arranging transnational consanguineous marriage was a key way to increase the power and status of one’s biraderi. A man who cannot produce children thus lets down his biraderi, and his share of inheritance may go to rival kin. One British-born woman in her mid-thirties, undergoing treatment to conceive a second child, described how these issues play out when a man is childless:

We go to Pakistan every year... and it’s even worse over there, everybody feels they can say to you ‘oh, you’ve got no children’... ‘it’s so sad and look at your big house and there is nobody to inherit it’ and you know they really make you feel bad and the same with my husband, his friends and [their] children and he is likely to go to a shop and buy something for cousins and their children and it will be ‘ah, it’s because he’s got no children of his own’… so you know it’s really hard…

Some men still showed a strong commitment to their conjugal relationships, even in the absence of their own biological children. This may explain some cases of disengagement or reluctance to provide semen samples. Hasan, a British-born man in his thirties, explained:

I didn’t want to think or blame one another. I held back to be honest because I didn’t want anything negative to come out and put pressure on my wife or myself. I wanted to look at all parts to it. I didn’t think that I would go to the medical and expect everything to be hunky dory. It just doesn’t work like that. You have to look at these things negatively as well. This is because if things don’t turn out how you would like them to at least you are prepared for the things you would want to possibly hear as you are going to go to hear some sort of news. To be honest with my wife it was … a little bit different. She did want to hear something positive, but I had to consider other possibilities. It wasn’t my idea in the first place to go to the medical to be honest. But my wife insisted we go through it… I also think because of my positive attitude, it kept us going. But she was very upset a few times. I think it made us closer if anything else… It actually showed how strong we were as a couple and showed others around us. We didn’t argue or criticize one another in anyway and I reassured that I was 100 percent happy with her.

Hasan explained his skepticism about IVF, based on observations of other family members suffering many unsuccessful cycles. However, their consultant had simply assumed that he and his wife would want IVF and had placed them on the waiting list. Concerned about the stress and the high chance of IVF failure, Hasan and his Pakistan-born wife decided to ignore the appointment letter when they reached the top of the waiting list, and instead opted to adopt a child. Hasan had no complaints about the clinical staff in the hospital: “The staff were brilliant to us, but, again, automatically assumed that we would consider IVF as the right course of treatment.” Hasan’s reluctance to have anything negative emerge about him or his wife made him hesitate about diagnosis, lest the one without the problem be pressured to leave the other. Consequently, disengagement may have different meanings in different cases, and may not necessarily indicate discord or a lack of commitment to the relationship.

There was agreement across all interviewees that, amongst Pakistani couples, women are typically blamed for fertility problems and that male factor infertility was considered to be an afterthought, when it became obvious, or was eventually diagnosed. In several interviews with people who had not faced infertility, there was laughter when I mentioned that infertility might be a man’s fault. Stories of men hiding known male factor infertility by taking second wives were also shared.

The temporal dimension of the infertility experience is crucial. Woman-blaming is the first step, and men have more room to maneuver early on in treatment, before male factor infertility is diagnosed. Some men may have disengaged from clinical contact to protect their masculinities and deflect blame on to their wives. However, as the case of Hasan shows, men may be mindful of the balance of risk to benefit in their conjugal relationships of engaging with NHS treatment.

Not all men were interested or able to maintain strong relationships despite infertility, and several divorced middle-aged women told me they had been left by their husbands because of infertility. The idea that the purpose of marriage was to produce children was taken as self-evident, and divorce or new marriage was considered by some men and women to be a logical response to persisting infertility. Asif, a Pakistan-born man in his fifties with several children, explained how he came to marry his second wife:

[My first wife had] seen the doctors after a couple of years and the doctors turned around and said there’s no chance … and then it dragged along for about ten years … only my father said, well, you know what fathers are like, and with me being the only son, they wanted kids … you know grandchildren so you know it was one of them things … They wanted us to do something about it… [my first wife] stood by me all the way, and a good job she stand by me because now what’s happened is the kids live with her and [my second wife] lives separately … So I mean the main thing for a mother is to be called a mother.

Author: Yeah.

Asif: And that’s a big thing and when you’ve got, she’s got kids now and nobody can tell that or nowt’ because they’re like that [gestures to show two fingers very close together] with her now like all the time.

Author: Right and they call her *ami* [mom] and everything?

Asif: Yeah.

Asif explained that he had not gotten along well with his second wife, and so they were living apart, and he was living with his children and his first wife. This suggests that a couple’s strong commitment to parenting and to the conjugal relationship can take more than one form.

Several women explained that they bore the burden of blame more or less willingly to protect their husbands. Naseem, for instance, could have revealed her suspicions of male infertility to her inlaws to dissuade them from harassing her, but she chose not to and angrily confronted her husband instead. In other cases, women praised and protected their husbands, clearly committed to the conjugal relationship and choosing not to reveal the cause of infertility because “it would be degrading to your husband.” Walle (2004) has written about the way in which maintaining secrecy around things which might cause shame provides protection for Pakistani men; families comply with this as it is in their interest as well, but this extends beyond Pakistani men, including to white British men (see Throsby and Gill 2004).

## Conclusions

Understanding the broader context of British Pakistani men’s lives can illuminate the perceptions of health professionals with whom they engage in fertility treatment settings. For British Pakistanis, infertility is a highly stigmatized condition for which women typically take the blame, regardless of the cause, in order to protect men. Similar findings about the gendered nature of infertility stigma have derived from studies across a broad geography (Inhorn and Van Balen 2002; Inhorn 2003; Culley and Hudson 2007). Tendencies to blame women can be disrupted by technologies that allow male factor infertility to be easily diagnosed (Parrott 2014), but this may be actively resisted. Health care systems that view male factor infertility diagnosis as a necessary stage in treatment protocols can assist more couples to access advanced treatments, but this too can be resisted in order to protect men (Bennett 2012). Further, men sometimes choose to disengage from treatment, either to protect themselves or their relationships.

Apparent expressions of anger and resistance to diagnoses of male infertility may mask other forms of emotional distress, known to wives but not to clinicians. The perception of this group of men as “angry” may relate to a long and continuing history of stereotyping South Asian Muslim men as dangerous. Of course, British Pakistanis are not a singular, bounded population, nor is there only one way to be a British Pakistani man. As mentioned above, many of the concerns of the couples I interviewed would be common to patients of other ethnic backgrounds. However, some of the contextual factors shaping the lives of British Pakistanis and people belonging to other ethnic groups may differ, including the role of kin and the impact of Islamophobia. Additionally, how men’s actions are interpreted by others, particularly by health practitioners, are likely to differ. As I have explored, Pakistani men find themselves in a subordinated position with respect to UK hegemonic masculinities, which leaves them more vulnerable to the failure to achieve masculine roles such as fatherhood that is highly valued according to both British and Pakistani norms. Pakistani men’s public responses to experiencing infertility, which are characterized by disengagement, appear to be in line with Pakistani social expectations of how men should behave, that is, silently and stoically, without expressing their vulnerability.

This raises questions about the effects of enforcement of a particular form of conjugal relationality, and about narrow cultural ideas about “genuine marriage,” on British Pakistani couples affected by infertility. These results disrupt the assumption that conjugal relationships are and must be based on one set of relational and emotional dynamics in order to be understood as constituting a valid marriage. Given the negative attention on Pakistani marriage practices by the British government (Charsley and Benson 2012) and the NHS (Shaw 2009), and prior evidence of racism and/or Islamophobia in the NHS (Bowler 1993; Coker 2001; Laird et al. 2007), such interpretations of what constitutes legitimate marriages take on the character of discrimination. An appreciation of and respect for different cultural norms of conjugality may well assist hospital staff to interact with Pakistani couples in more positive and productive ways in the UK context. If clinical judgment in the UK is expected to extend to “take account of wider family and social networks,” perhaps it should also consider the multiple ways for a family to demonstrate a capacity for supportive parenting.

## Footnotes

1 These government run centers offer services, including childcare and education, for deprived children and their families.

2 Snowball sampling is a non-probability sampling technique in which participants assist the researcher in recruiting further participants by using their social networks.

3 The UK government calculates its indices of deprivation based on seven domains of deprivation: income, employment, education, health, crime, barriers to housing and services, and quality of the living environment.
